# Genotype-phenotype correlations and expansion of the molecular spectrum of *AP4M1-*related hereditary spastic paraplegia

**DOI:** 10.1186/s13023-017-0721-2

**Published:** 2017-11-02

**Authors:** Conceição Bettencourt, Vincenzo Salpietro, Stephanie Efthymiou, Viorica Chelban, Deborah Hughes, Alan M. Pittman, Monica Federoff, Thomas Bourinaris, Martha Spilioti, Georgia Deretzi, Triantafyllia Kalantzakou, Henry Houlden, Andrew B. Singleton, Georgia Xiromerisiou

**Affiliations:** 1 Department of Molecular Neuroscience, Institute of Neurology, University College London, London, WC1N 3BG UK; 2 Department of Clinical and Experimental Epilepsy, Institute of Neurology, University College London, London, WC1N 3BG UK; 3 Department of Neurology, Medical State University N, Testemitanu, Chisinau, Moldova; 4 Laboratory of Neurogenetics, National Institute on Aging, National Institutes of Health, Bethesda, MD 20892 USA; 5 Department of Neurology, Papageorgiou Hospital, Thessaloniki, Greece; 6 Neurology Department of Aristotle University of Thessaloniki, AHEPA University Hospital, Thessaloniki, Greece; 7 National Hospital for Neurology and Neurosurgery, University College London Hospitals, London, WC1N 3BG UK

**Keywords:** Whole exome sequencing, AP4 complex, Epilepsy, Hereditary spastic paraplegia, Cerebellar hypoplasia

## Abstract

**Background:**

Autosomal recessive hereditary spastic paraplegia (HSP) due to *AP4M1* mutations is a very rare neurodevelopmental disorder reported for only a few patients.

**Methods:**

We investigated a Greek HSP family using whole exome sequencing (WES).

**Results:**

A novel *AP4M1A* frameshift insertion, and a very rare missense variant were identified in all three affected siblings in the compound heterozygous state (p.V174fs and p.C319R); the unaffected parents were carriers of only one variant. Patients were affected with a combination of: **(a)** febrile seizures with onset in the first year of life (followed by epileptic non**-**febrile seizures); **(b)** distinctive facial appearance (e.g., coarse features, bulbous nose and hypomimia); **(c)** developmental delay and intellectual disability; **(d)** early**-**onset spastic weakness of the lower limbs; and **(e)** cerebellar hypoplasia/atrophy on brain MRI.

**Conclusions:**

We review genotype-phenotype correlations and discuss clinical overlaps between different AP4-related diseases. The AP4M1 belongs to a complex that mediates vesicle trafficking of glutamate receptors, being likely involved in brain development and neurotransmission.

**Electronic supplementary material:**

The online version of this article (10.1186/s13023-017-0721-2) contains supplementary material, which is available to authorized users.

## Background

Clinically and genetically heterogeneous neurological disorders constitute huge challenges for clinicians and geneticists as the number of genes associated with a wide range of overlapping phenotypes is constantly increasing. For the molecular diagnosis of these disorders gene-by-gene screens are progressively being replaced by more time and cost effective next generation sequencing approaches (candidate gene panels or whole-exome sequencing) [1, 2]. Hereditary spastic paraplegias (HSPs) are an example of such a heterogeneous group of disorders, for which more than 70 loci have already been mapped and yet the landscape of HSP loci and genes is far from complete [3]. HSPs are characterized by progressive spasticity and weakness of the lower limbs due to corticospinal tract dysfunction. HSPs are broadly classified as uncomplicated or complicated on the basis of the presence of additional clinical features such as intellectual disability, seizures, ataxia, peripheral neuropathy and visual defects [4].

HSP-associated genes are involved in a wide variety of primary molecular functions, resulting for example in disturbances in vesicle formation and membrane trafficking including selective uptake of proteins into vesicles when such genes are mutated [4]. This is the case for the subunits of the heterotetrameric adaptor protein complex 4 (AP-4). AP-4 is composed of two large chains beta-4 (*AP4B1*; MIM#607245) and epsilon-4 (*AP4E1*; MIM#607244), a medium mu-4 chain (*AP4M1*; MIM#602296), and a small sigma-4 chain (*AP4S1*; MIM#607243).

AP-4 complex-mediated trafficking is thought to play a crucial role in brain development and functioning. All genes encoding for the proteins part of this complex have been associated with genetic forms of HSP (e.g. *AP4B1* [SPG47], *AP4M1* [SPG50], *AP4E1* [SPG51], and *AP4S1* [SPG52]) [4, 5]. We hereby report on a Greek family with SPG50 (MIM#612936) caused by two heterozygous *AP4M1* variants in a compound heterozygous state. We also discuss on the genotype-phenotype correlations and the spectrum of AP4-related diseases.

## Methods

### Subjects

**Fig. 1:**
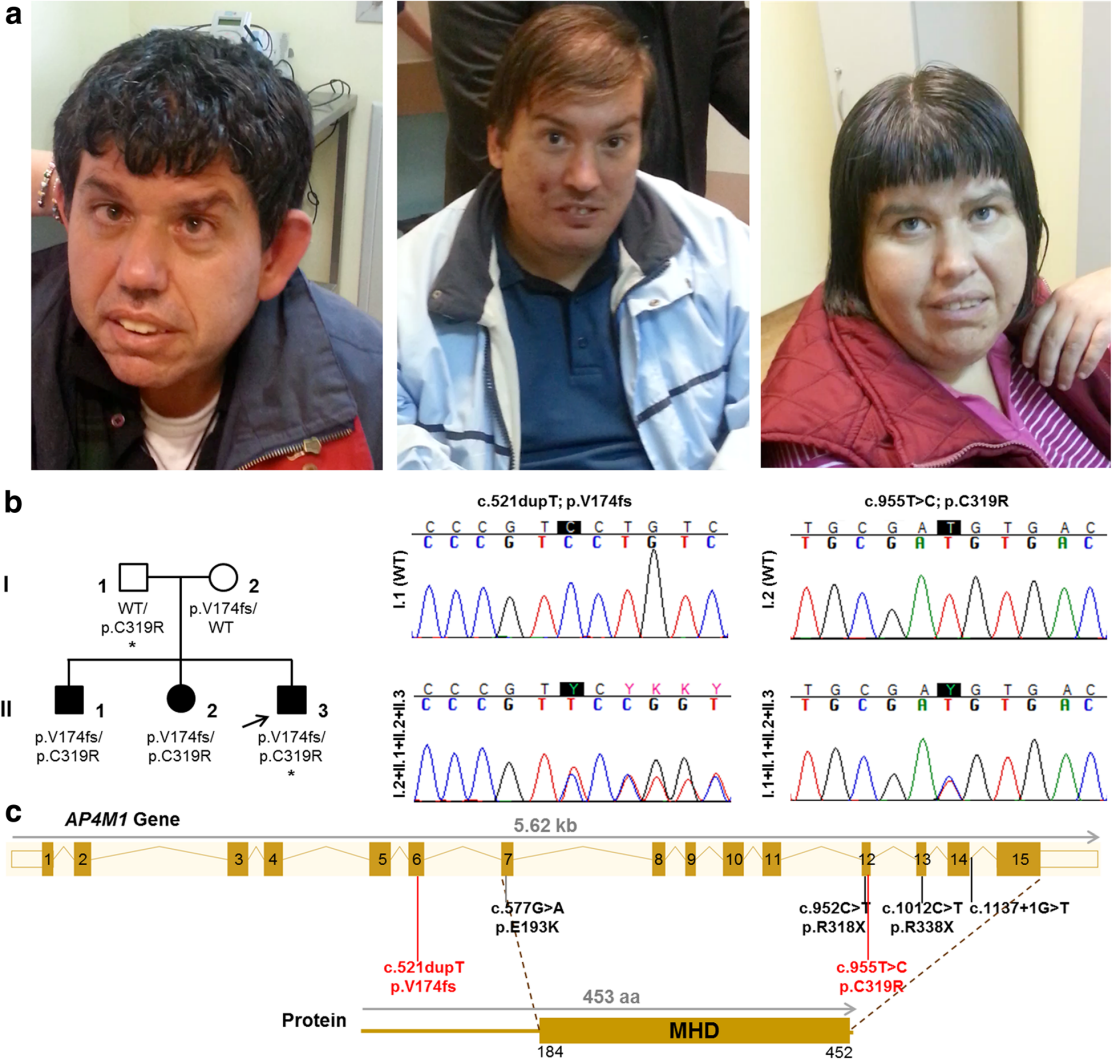
The three siblings affected by complicated HSP due to *AP4M1* compound heterozygous mutation. **a** Note facial coarse features, convergent bilateral strabismus, prominent and bulbous nose, and wide mouth in all the three siblings; (**b**) In the left panel familial pedigree (the asterisks specify the sequenced individuals with the arrow pointing to the proband) with black and white symbols indicating affected and unaffected individuals, respectively; in the right panel electropherograms illustrating the *AP4M1* variants identified in our family; (**c**) A schematic representation of the *AP4M1* gene and protein depicting the location of the mutations found in our family (in red) and those previously reported in the literature (in black) for this gene (MIM#602296), and highlighting the MHD, which is a protein-protein interaction module. HSP = *Hereditary Spastic Paraplegia*. MHD = *mu homology domain*

We have studied a Greek family (Fig. 1) composed of three affected siblings born to healthy parents, who presented with complicated HSP. History of previous neurological disease was unremarkable in the family and the pedigree suggested an autosomal recessive inheritance. Thorough neurological examination and follow-up were carried out at the Departments of Neurology of the AHEPA Hospital and Papageorgiou Hospital by some of the authors (GX, TK, GD, MS, TB and HH). This study was approved by the UCLH institutional-research-board. After informed consent, we collected blood samples from the patients and their parents, and extracted DNA using standard procedures. Additional informed consent was obtained from all individual participants for whom identifying information is included in this manuscript.

### Genetic analysis

To investigate the genetic cause of the disease in this family, whole-exome sequencing (WES) was performed in the proband (Fig. 1b: II-3) and his father (Fig.1b: I-1). Nextera Rapid Capture Enrichment kit (Illumina) was used according to the manufacturer instructions. Libraries were sequenced in an Illumina HiSeq3000 using a 100-bp paired-end reads protocol. Sequence alignment to the human reference genome (UCSC hg19), and variants call and annotation were performed using an in-house pipeline as described elsewhere [6]. The raw list of single nucleotide variants (SNVs) and indels was then filtered. Only exonic and donor/acceptor splicing variants were considered. In accordance with the pedigree and phenotype, priority was given to rare variants [<1% in public databases, including 1000 Genomes project, NHLBI Exome Variant Server, Complete Genomics 69, and Exome Aggregation Consortium (ExAC v0.2)] fitting a recessive model (i.e. homozygous in the proband but heterozygous in the father or compound heterozygous in the proband but not in the father), and located in genes previously associated with HSP [3, 7].

The *AP4M1*A mutations (in exons 6 and 12) identified by WES in the proband were confirmed by traditional Sanger sequencing and also segregation analysis of the mutation in the family was performed. Detailed conditions of sequencing analysis are available upon request.

## Results

### Clinico-radiological phenotype

The three affected siblings (Fig. 1b: II-1, II-2 and II-3) have a phenotype consisting of complicated HSP. All of them showed early-onset and severe spastic lower limb weakness, brisk deep tendon reflexes, presence of Babinski sign and severe gait difficulties. The older male and the female (Fig. 1b: II-1 and II-2, respectively) need assistance to stand up. Only the younger male (Fig. 1b: II-3) is able to stand up independently and walk a few steps unassisted. The gait is characterized by feet dragging, shaking and leg scissoring. All three siblings have upper limbs weakness, more obvious in the proximal muscle groups, especially for the two older siblings. The facial muscles are hypotonic with reduced facial expressions. The facial appearance of the three siblings is characterized by coarse features, a prominent and bulbous nose and a wide mouth (Fig. 1a; left panel patient II-1, middle panel patient II-3, right panel patient II-2).

Interestingly, all affected siblings present hypometric and slow vertical saccades, especially at the upward gaze. Some limb ataxia is also present. There are no prominent extrapyramidal signs.

All three siblings have severe intellectual disability. Additional cognitive/behavioral abnormalities include apathy, reduced motor planning and/or initiation, attention-deficit disorder. An impairment of speech is also present, especially for the older male who has very poor language skills.

All siblings were born without any adverse perinatal events and had normal development during the first months of their lives. All three presented febrile tonic-clonic seizures during the first year of life, which were soon followed by epileptic non-febrile seizures. Developmental delay was noticed since early infancy. Only the younger brother (Fig. 1b; patient II-3) managed to walk independently during childhood but later his motor skills also started to gradually decline. The language and social skills of all three siblings were also lagging behind during their childhood and eventually showing some serious deficits by their puberty.

Spastic Paraplegia Rating Scale (SPRS) [8] was assessed for all 3 siblings: the older male and the female (Fig. 1b: II-1 and II-2, respectively) both scored 40 out of a maximum of 52, while the younger male (Fig. 1b: II-3) scored 35/52.

Other disorders presenting with spastic paraparesis (e.g. abetalipoproteinemia, funicular myelosis, multiple sclerosis, AIDS, lues, adrenoleucodystrophy, etc.) were excluded after an extensive diagnostic work-up that included brain magnetic resonance imaging (MRI), cerebrospinal fluid (CSF) analysis, nerve conduction and evoked potentials studies, electromyography (EMG), electroencephalography (EEG), serum vitamin B12 and E levels, serum arylsulphatase, galactocerebrosidase and very long chain fatty acids (VLCFA) levels, HTLV-1 antibodies, HIV and syphilis serology.

**Fig. 2:**
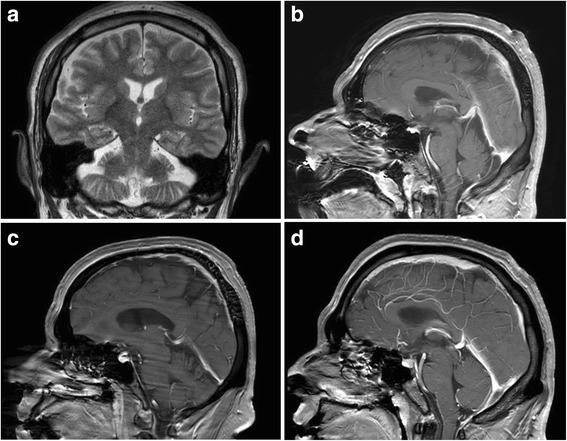
Brain MRI of the three affected siblings. **a** T2-weighted coronal scan of the older male patient (Fig. 1b: II-1) demonstrates cerebellar hypoplasia/atrophy with the impression of a cleft in the lateral cerebellar hemisphere. Note that the brainstem volume is preserved; (**b**, **c**, **d**) T1-weighted sagittal scans with contrast of all three siblings (Fig. 1b: II-1, II-2 and II-3, respectively) show slight hypoplasia/atrophy of the cerebellar vermis. Also, there is downsloping splenium of the corpus callosum

The brain MRI showed cerebellar hypoplasia/atrophy and downsloping splenium of the corpus callosum (Fig. 2).

Nerve conduction studies showed no evidence of peripheral neuropathy. Somatosensory evoked potentials (SSEPs) of median and tibial nerve were remarkable for prolongation of cortical latencies (N13-N20 and P40, respectively).

EEG was reported normal for the three siblings, without findings of focal slowing or seizure-like activity. More specifically, EEG recordings showed background of 9 to 10 hertz alpha activity, maximal over the posterior head region. These activities were symmetric on both sides and attenuated with eye opening. No focal slowing was seen and no seizure-like activity was observed during the recording. Patient entered into periods of drowsiness and light sleep. No abnormality was seen.

All clinical and radiological features are summarized in Table 1, and compared to other cases of HSP due to *AP4M1* mutations previously reported in literature.

**Table 1:** Summary of clinical and radiological features of patients with *AP4M1* mutations

		Present study	Τüysüz et al., 2014 [5]		Verkerk et al., 2009 [13]	Jameel et al., 2014 [14]	Langouet et al., 2015 [15]
Ethnic Background		Greek	Turkish	Turkish	Moroccan	Pakistani	Algerian
Pedigree		2 M/1F	2F	1 M/1F	3M/2F	2M	1 M/1F
Intellectual disability		Severe	Severe	Moderate to severe	Severe	Severe	Severe
Speech impairment		+	+	+	+	+	+
Pseudobulbar signs (e.g. stereotypical laughter)		–	+	+	+	–	+/−
Infantile hypotonia		NA	NA	+	+	+	+
Seizures		+	+	+	–	+/−	+/−
Spastic tetraplegia		+	+	+	+	–	+
Hypertonia		+	+	+	+	+	+
Hyperreflexia		+	NA	NA	NA	+	+
Babinski sign		+	NA	NA	+	+	+
Loss of ambulation		+	+	+	+	+	+
Ataxia		+	NA	NA	–	NA	NA
Slow/hypometric saccades		+	NA	NA	NA	NA	NA
Facial hypotonia		+	+	+	NA	–	NA
Coarse facial features (e.g. bulbous nose)		+	+	+	NA	+/−	–
MRI features	Ventriculomegaly	–	+	+	+	+	+
	Thin splenium	+	+	+	+	+	+
	White matter abnormalities	–	–	+	+	–	+
	Cerebellar atrophy	+	–	–	+	+	–
Mutation		c.521dupT/c.955 T > C	c.1012C > T	c.952C > T	c.1137 + 1G > T	c.194_195delAT	c.1137 + 1G > T

*M* Male, *F* Female, *NA* Not available; +: Present; −: Absent; +/−: Present only in some members of the family

### Sequence analysis

WES generated a total of 136,968,572 (proband, Fig. 1b: II-3) and 145,150,172 (father, Fig. 1b: I-1) unique reads, with an average on target depth over 160 reads, and >98% of the target bases covered at least 10X. A total of 24,872 (proband) and 25,228 (father) exonic/splicing variants were detected. After applying our filtering strategy described above, we identified two heterozygous *AP4M1* (SPG50) variants in the proband, a novel frameshift insertion (c.521dupT; p.V174fs in exon 6), and a very rare (<2.5 × 10^−5^ in public databases) missense variant (c.955 T > C; p.C319R in exon 12). Only one of these variants was present in the father (c.955 T > C; p.C319R). No other rare homozygous or compound heterozygous variants were found in genes of relevance for the phenotype, including all know HSP genes. The *AP4M1*frameshift variant causes a very premature stop codon, truncating the protein (if expressed at all) to 179 amino acids (wild-type: 453 amino acids), just before the mu homology domain (MHD) which is an essential protein-protein interaction module (Fig. 1c). The missense variant lays on a highly conserved position (GERP++ score [9] of 4.75) of the MHD domain, and is predicted to be damaging by several prediction tools (including SIFT [10], PolyPhen2 [11], and MutationTaster [12]). Segregation analysis by Sanger sequencing showed that all three affected siblings have both variants, whereas the father carries only the missense and the mother carries only the frameshift (Fig. 1b), confirming that the compound heterozygous state of these variants is segregating with the phenotype.

## Discussion

Very few patients and families have been described in the literature with mutations in the *AP4M1* gene. The reported phenotype is mainly characterized by the combination of infantile hypotonia, developmental delay, intellectual disability, early-onset spastic paraplegia, and variable white matter and cerebellar involvement on brain MRI; only four pathogenic *AP4M1* mutations (Fig.1c) have been reported to date in SPG50 patients [5, 13–17].

Our pedigree is compatible with an autosomal recessive complicated spastic paraplegia, and the two variants we identified in the *AP4M1* gene, segregating with the phenotype, are very likely the cause of the phenotype in this family. Copy number variants (CNVs) involving the *AP4M1* gene have also been implicated in developmental disabilities or congenital anomalies [18, 19]. The *AP4M1* gene is highly expressed in the brain especially during foetal development (see Additional file 1: Figure S1), and disruption of its function compromises proper brain development and likely impairs neuronal excitability.

Notably, patients with biallelic loss-of-function mutations of *AP4M1* have been initially reported with a phenotype presenting at birth with severe infantile hypotonia and diffuse white matter loss on brain MRI. For this reason it has been postulated that the genetic defect in these patients results in abnormal cycling of glutamate receptors, mimicking glutamate-mediated perinatal white matter injury [13]. Interestingly, the affected siblings from our family showed a milder presentation, with normal muscular tone during their infancy and no white matter involvement on their brain MRI, similarly to the other few patients reported with recessive missense mutations in *AP4M1* [5]. It is therefore possible that in our cases the missense mutation inherited on the paternal allele resulted in a milder phenotype because of a residual function of the gene, compared to the most severe cases where homozygous splicing mutations have been identified as the cause of the disorder [13].

Interestingly, the earliest manifestation in the natural history of the disease in our patients were tonic-clonic seizures (precipitated by fever), which appeared since the first months after birth. Of note, febrile seizures with onset in the first year of life represent common features of *AP4B1* deficiency, an overlapping (autosomal recessive) HSP phenotype (SPG47; MIM#614066) caused by mutations in the gene encoding the large b4 chain of the AP-4 complex [20].

It has been shown that the loss or structural change of a single AP-4 subunit impairs the integrity of the entire AP-4 complex [21]. Consequently, mutations in any of the AP-4 subunits would presumably have similar downstream effects on vesicular glutamate receptor transport and neurotransmission, resulting in similar clinical presentations (e.g., altered neuronal excitability and risk of developing infantile febrile seizures in both *AP4B1-* and *AP4M1* **-**related phenotypes). These observations are further corroborated by the few patients reported with recessive missense mutations in *AP4M1,* who also presented seizures (precipitated by fever) during their first year of life [5].

## Conclusions

We reported on genotype-phenotype correlations in SPG50, basing on a Greek family with three affected individuals and the few previously reported patients, and also expanded the molecular spectrum associated with this phenotype. Further studies will be needed to investigate the role of AP**-**4 in brain development and neurotransmission and to fully understand the pathophysiology of childhood epilepsy in these patients.

## Additional file

Additional file 1: FigureS1. Expression of the *AP4M1* gene in several regions of the human brain throughout development and aging. Note the higher expression levels during fetal development (birth is marked with a vertical solid line). Data from the Human Brain Transcriptome (HBT) project (http://hbatlas.org). CBC - cerebellar cortex, MD - mediodorsal nucleus of the thalamus, STR - striatum, AMY - amygdala, HIP - hippocampus, and NCX – neocortex. (PDF 68 kb)

## Abbreviations

AIDS: Acquired immune deficiency syndrome
AP-4: Adaptor protein complex 4
CNVs: Copy number variants
CSF: Cerebrospinal fluid
DNA: Deoxyribonucleic acid
EEG: Electroencephalography
EMG: Electromyography
ExAC: Exome Aggregation Consortium
GERP++: Genomic Evolutionary Rate Profiling
HIV: Human immunodeficiency virus
HSP: Hereditary spastic paraplegia
HTLV-1: Human T-cell lymphotropic virus type 1
Indels: Small insertions and deletions
MHD: Mu homology domain
MIM: Online Mendelian Inheritance in Man database reference number
MRI: Magnetic resonance imaging
NHLBI: National Heart, Lung, and Blood Institute from the National Institutes of Health
PolyPhen2: Polymorphism Phenotyping v2 algorithm
SIFT: Sorting tolerant from intolerant algorithm
SNVs: Single nucleotide variants
SPG47: Spastic paraplegia type 47
SPG50: Spastic paraplegia type 50
SPG51: Spastic paraplegia type 51
SPG52: Spastic paraplegia type 52
SPRS: Spastic paraplegia rating scale
SSEPs: Somatosensory evoked potentials
VLCFA: Very long chain fatty acids
WES: Whole-exome sequencing
