# Supplementary material for: Genotype-phenotype correlations and expansion of the molecular spectrum of AP4M1-related hereditary spastic paraplegia
Source: Orphanet J Rare Dis. 2017 Nov 2;12:172. doi: 10.1186/s13023-017-0721-2 (PMC5669016; doi:10.1186/s13023-017-0721-2)
Supplement: Additional file 1: FigureS1. — Expression of the AP4M1 gene in several regions of the human brain throughout development and aging. Note the higher expression levels during fetal development (birth is marked with a vertical solid line). Data from the Human Brain Transcriptome (HBT) project (http://hbatlas.org). CBC - cerebellar cortex, MD - mediodorsal nucleus of the thalamus, STR - striatum, AMY - amygdala, HIP - hippocampus, and NCX – neocortex. (PDF 68 kb) [file 13023_2017_721_MOESM1_ESM.pdf]

Bettencourt et al., **Genotype-Phenotype correlations and expansion of the molecular spectrum of *AP4M1*-related Hereditary Spastic Paraplegia**

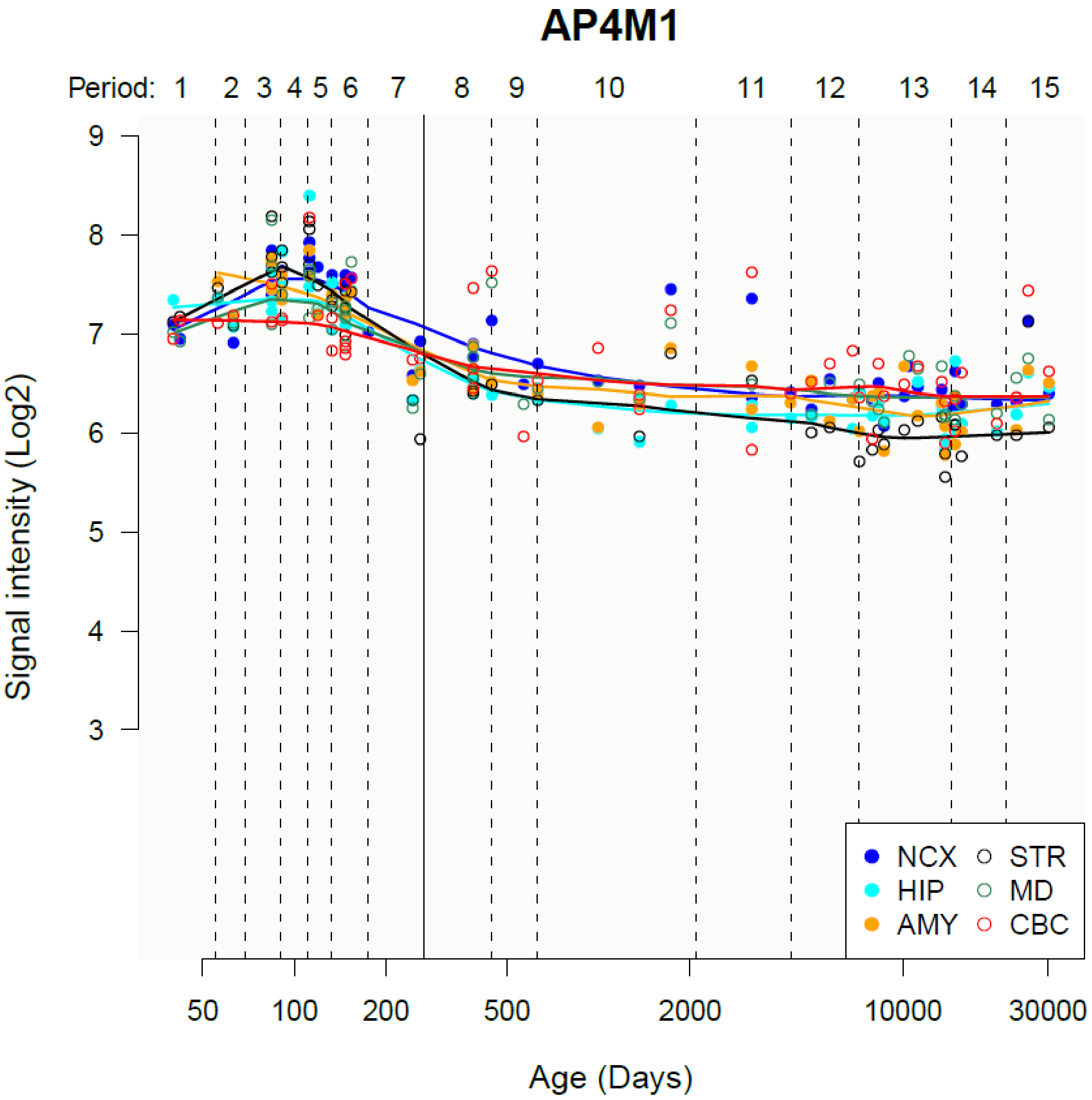

Supplementary Figure 1. Expression of the *AP4M1* gene in several regions of the human brain throughout development and aging. Note the higher expression levels during fetal development (birth is marked with a vertical solid line). Data from the Human Brain Transcriptome (HBT) project (http://hbatlas.org).

CBC - cerebellar cortex, **MD** - mediodorsal nucleus of the thalamus, **STR** - striatum, **AMY** - amygdala, **HIP** - hippocampus, and **NCX** - neocortex
